# Characterized non-transient microbiota from stinkbug (*Nezara viridula*) midgut deactivates soybean chemical defenses

**DOI:** 10.1371/journal.pone.0200161

**Published:** 2018-07-12

**Authors:** Virginia Medina, Pedro M. Sardoy, Marcelo Soria, Carlos A. Vay, Gabriel O. Gutkind, Jorge A. Zavala

**Affiliations:** 1 Universidad de Buenos Aires, Facultad de Agronomía, Cátedra de Bioquímica -Instituto de Investigaciones en Biociencias Agrícolas y Ambientales (INBA-CONICET), Buenos Aires, Argentina; 2 Universidad de Buenos Aires, Facultad de Agronomía, Cátedra de Microbiología -Instituto de Investigaciones en Biociencias Agrícolas y Ambientales (INBA-CONICET), Buenos Aires, Argentina; 3 Universidad de Buenos Aires, Facultad de Farmacia y Bioquímica, Buenos Aires, Argentina; 4 Consejo Nacional de Investigaciones Científicas y Técnicas de Argentina, (CONICET), Buenos Aires, Argentina; Institute of Plant Physiology and Ecology Shanghai Institutes for Biological Sciences, CHINA

## Abstract

The Southern green stinkbug (*N*. *viridula*) feeds on developing soybean seeds in spite of their strong defenses against herbivory, making this pest one of the most harmful to soybean crops. To test the hypothesis that midgut bacterial community allows stinkbugs to tolerate chemical defenses of soybean developing seeds, we identified and characterized midgut microbiota of stinkbugs collected from soybean crops, different secondary plant hosts or insects at diapause on *Eucalyptus* trees. Our study demonstrated that while more than 54% of *N*. *viridula* adults collected in the field had no detectable bacteria in the V1-V3 midgut ventricles, the guts of the rest of stinkbugs were colonized by non-transient microbiota (NTM) and transient microbiota not present in stinkbugs at diapause. While transient microbiota *Bacillus* sp., *Micrococcus* sp., *Streptomyces* sp., *Staphylococcus* sp. and others had low abundance, NTM microbiota was represented by *Yokenella* sp., *Pantoea* sp. and *Enterococcus* sp. isolates. We found some isolates that showed *in vitro* β-glucosidase and raffinase activities plus the ability to degrade isoflavonoids and deactivate soybean protease inhibitors. Our results suggest that the stinkbugs´ NTM microbiota may impact on nutrition, detoxification and deactivation of chemical defenses, and *Enterococcus* sp., *Yokenella* sp. and *Pantoea* sp. strains might help stinkbugs to feed on soybean developing seeds in spite of its chemical defenses.

## Introduction

Even if developing soybean seeds respond to stinkbug damage up-regulating important defenses against herbivore insects, as cysteine proteases inhibitors and isoflavonoids production [1,2], these induced chemical defenses are not sufficient to stop attack by the southern green stinkbug (*N*. *viridula*) or the red banded stinkbug (*Piezodorus guildinii*), what makes them the most harmful pests to soybean crops [3,4].

*N*. *viridula* has an annual life cycle that generally comprehends five generations [5]. During the onset of bloom and podset in late summer, soybean becomes attractive to stinkbugs, and the third generation of adults migrates into the crop. Subsequently, the fourth and fifth generations also develop within this crop, at a time when they can feed on their developing seeds [5]. However, when soybean (primary host) is not available stinkbugs specific preference for soybean pods changes to many other plant species depending on its maturity and phenology (secondary hosts: SH), and plants in stage of fruit and pod formation are more attractive [6]. Whereas in tropical or subtropical areas with mild winters *N*. *viridula* feeds on any secondary hosts available [6], in colder areas the last generation to reach adult stage seeks shelter under the bark of trees beginning diapause, which is a critical period for the population [7].

Diversification and evolutionary success of insects have depended in part on a number of relationships with beneficial microorganisms that have been known to increase the nutritional value of diets and allow the digestion of recalcitrant compounds [8–11]. Several studies have discussed the potential effects of diets on gut microbial composition and insect host [12–17]. A study comprehending 62 insect species, including *N*. *viridula*, showed that diet certainly affects intraspecific gut bacterial community profiles when the host and microbiota are intimately associated, such as in lignocellulose digestion [18]. Moreover, gut bacterial diversity is significantly higher in omnivorous than in stenophagous (carnivorous and herbivorous) insects [19]. It has been suggested that technologies used in agricultural production systems, such as changes in soil management by crop rotations or use of agrochemicals like pesticides and herbicides, may shape the microbial communities of soil and plants, inducing insects to adopt these new microbial species, which may help them to adapt to these altered or changing environments [20].

Symbiotic/aposymbiotc studies performed with two related stinkbug species (*Megacopta punctatissima* and *M*. *cribaria*) suggest that these species success as soybean crops pests is more related to their relationship with their obligate gut symbiont (*Ishikawaella capsulate*) than to specific traits of the insect species [21]. Another interesting example is the variant of the corn (*Zea mays*) pest western corn rootworm (WCR; *Diabrotica virgifera virgifera Le Conte*) which in last years started feeding on soybean foliage and also acquired tolerance against cysteine protease inhibitors, a specific defense against Coleopteran insects [22]. Gut microbiota analysis of the new variant of WCR suggests that it is the bacterial community what allows the insects to tolerate defenses and to feed on the new host (soybean) [23]. Although the cyclic annual feeding behavior and host multiplicity of *N*. *viridula* could have some impact on gut microbial community composition, it seems clear that understanding the impact of feeding on developing soybean seeds on microbial community may explain stinkbugs behavior.

Previously, it has been shown that *Klebsiella pneumoniae* and *Enterococcus faecalis* are present in the midgut of *N*. *viridula* specimens collected in Brazil [24]. Moreover, *Klebsiella pneumoniae* was isolated from *N*. *viridula* adults collected in soybean fields near College Station, TX [25]. Other studies have focused on an obligate symbiont resident in the caeca of *N*. *viridula* that may have relevance on nymph survival, which has been consistently identified in Brazil, Hawaii, California and Japan [24,26–28]. Egg-surface sterilization disrupts nymphal infection with the symbiont, indicating vertical transmission of the gut symbiont via egg surface contamination[26]. Eliminating the symbiont resulted in severe nymphal mortality and emergence of few adult insects. These results contrasts with those studies on Hawaiian populations of *N*. *viridula* [27,29], wherein elimination of the gut symbiont caused few fitness defects in the host. Although *Streptomyces* sp strains were also associated with the caeca of lab reared stinkbug adults, no further analysis was performed [30]. In addition, a *Pantoea* sp. strain has been associated with the caeca of another stinkbug pest, *Halyomorpha halys* [31], and the lack of this symbiont decreases survivorship of stinkbug subsequent generations [32]. However, little is known about the biological function of the bacteria present in stinkbugs ventricles where digestion is performed (V1-V3 of the midgut)[33]. Since *N*. *viridula* is polyphytophagous and tolerate plant defenses, characterization of the bacterial community associated with insects collected in field production areas will improve our understanding of stinkbug-soybean interactions.

To study the mechanisms of midgut bacteria-insect symbiosis that might help stinkbugs to overcome plant defenses, we characterized the midgut microbial community potentially related with digestion and associated to different stinkbug’s hosts (diet) along the year. In addition, we determined diapause influence on stability and composition of resident microbiota. Finally, we characterized isolated bacteria and identified potential functional activities related to soybean digestion, and inactivation of chemical defenses, such as cysteine protease inhibitors. Our results allowed us to draw conclusions about the possible functions of midgut bacterial community in circumventing soybean defences by the southern green stinkbug in the main production areas of central Argentina.

## Materials and methods

### Sample collection and treatments

To assess variations of the microbial community composition in guts of stinkbugs collected across different geographical locations, 173 *N*. *viridula* (Hemiptera, *Pentatomidae*) adults were collected in 26 collecting events: 8 collections from different species of plants with the exception of soybean, which were considered as secondary hosts (SH; total of 52 stinkbugs), 9 collections from soybean crops (53 stinkbugs) and 9 from *Eucalyptus* trees (diapause; 68 stinkbugs) at different moments of the year with a random sampling design along three years (2012–2014). Sample collections events were distributed in 15 different sites located in central east Argentina: Rafaela (-31,269161–61,484985), Paraná (-31.866785, -60.483346) and Oliveros (-32,578063, -60,853958) (Santa Fé Province), Pincen (-34,834096, -63,923950) and Marcos Juarez (-32,699489, -62,100220) (Córdoba Province), La Plata (-35,014814, -58,069611), Lujan (-34,569906, -59,118805), Pergamino (-33,897777, -60,571060), Rojas (-34,198173, -60,731049), Carabelas (-34,037867, -60,871811), Pila (-35,980229, -57,994852), San Antonio de Areco (-34,265161, -59,449768), Chacabuco (-34,642247, -60,852295) and General Villegas (-35,056980, -63,006592) (Buenos Aires Province), and the experimental field of our Facultad de Agronomía, Universidad de Buenos Aires in Buenos Aires city (-34,590259, -58,457565) (S1 Table and S1 Fig). All collecting events were carried out on private land with the exception of one carried out on Facultad de Agronomía experimental fields that belong to our place of work. The owners of private lands gave permission to conduct de collecting of *Nezara viridula* and also gave information about pesticides applications. Field studies did not involve endangered or protected species. The funders had no role in study design, data collection and analysis, decision to publish, or preparation of the manuscript.

Insects were collected from soybean crops at reproductive stage or plant species around the crops, where pesticides were never applied before each collection event. Geographical location (latitude and longitude) of each site was registered by GPS and plant species where *N*. *viridula* adults were found, identified and annotated (S1 Table). Samples were composed by 4 to 10 adults of stinkbugs that were handpicked and dissected to analyze bacterial community. Gut community of stinkbugs was analyzed by Automated Ribosomal Intergenic Spacer Analysis (ARISA) supplemented with agar plate culturing techniques on Trypticase Soy Agar (TSA) media. Based on differences in colony morphology on TSA plates, 21 different isolates were preliminarily identified by 16S rRNA sequencing. Frequency and abundance of each identified bacteria were annotated with the aim of hierarchize its biological importance. For those isolates that needed more accurate identification MALDI-TOF MS bacterial identification technique was performed. Based on the ability of each bacterium to remain in the gut of stinkbugs during diapause, and their abundance and their frequency of appearance, we classified bacterium as either Non-Transient Microbiota (NTM) (bacterial count ≥ 10^4^ CFU/mg gut and present in SH, soybean and *Eucalyptus* trees) or Transient microbiota (TM) (bacterial count under 100 CFU/mg gut, present only in SH and soybean). Members of the NTM were characterized by API 20E for enterobacteria, and 50 carbon sources fermentation API 50CH strips (Biomerieux). Phylogenetic analysis was performed by comparing sequences obtained from the analysis of isolated bacteria with those of reference strains indexed at the LPSN site (http://www.bacterio.net/). Bacterial localization was analyzed through microdissection of midgut ventricles and ARISA (S2 Fig). To determine potential functionality of NTM bacteria isolated and identified from the gut of stinkbugs, *in vitro* metabolic assays, including lipolytic, proteolytic and glycolytic activities were performed. To assess the ability of bacteria to decrease inhibitory activity of soybean cysteine proteases inhibitors, soybean meal was fermented with NTM isolated bacteria and compared against cysteine protease papain as control.

### Insect dissection, bacteria isolation and DNA extraction

Stinkbugs were dissected under aseptic conditions no later than 8 h after collection. Guts were entirely removed from insects and pooled on 1000 μL sterile buffer phosphate pH 7, and disrupted by homogenization with a plastic pestle. For bacterial count and isolation, 100 μl aliquot were serially diluted and plated on Trypticase Soy Agar and cultured at 37 °C for 18 h under aerobic conditions, Colonies with morphological differences of 17 different agar plates (bacterial gut communities of individual insects) were chosen for further isolation, identification, ARISA chromatograms performance, and in vitro activities (S2 Table). Remaining homogenates of individual guts from each sample were pooled and total gut DNA was purified with PowerFecal DNA Isolation kit (MOBIO) to perform gut bacterial community analysis with ARISA (S2 Table).

### Frequency and abundance of bacteria

Frequency of each identified bacterium was defined as positive results on plate counts (isolated and identified) and/or ARISA detection. Relative abundance was the ratio between colony forming units in 1 mg of intestine (CFU/mg of gut) of each species of bacteria identified and the total number of colonies counted on the agar plate.

### Isolated bacteria identification

For initial characterization, Gram staining and oxidase test [34] were used. DNA extraction of each isolate was performed with UltraClean Microbial DNA Isolation Kit (MOBIO) and two independent 16S rRNA fragments were PCR-amplified and sequenced. Firstly, the variable region V4 was amplified with universal primers 530f (5´-GTGCCAGCMGCCGCGG-´3) and 1392r (5´-ACGGGCGGTGTGTRC-3´) according to Geib, SM et al (2009)[35]. For those isolates that needed more accurate identification, a near-full length 1,450 bp fragment of 16S rRNA was amplified according to Lehman, RM et al. (2009) [36]. PCR products were ligated into pGEM-T Easy vector (Promega), and *Escherichia coli* DH5a were transformed. Plasmids were extracted with a QIAprep^®^ Spin Miniprep kit (QIAGEN, Valencia, CA). Briefly, the inserts were amplified using the vector flanking sequences as primers (T7 and sp6 promoters). A second pair of internal primers Sp6L01 (5`-AGTTTAT CACTGGCAGTCTCC-3´) and T7H01 (5´-GTACTTTCAG CGAGGAGAAG-3´) were used for sequencing the central 700 bp region. The BioEdit Sequence Alignment Editor software was used to build the entire sequence. Sequencing was done at Leloir Institute Facility, Buenos Aires, Argentina. The complete 16S rRNA gene sequences of strains isolated in this study are sequences of known species previously isolated by other groups, and these sequences are available for electronic retrieval from the EMBL, GenBank.

All isolates were deposited in the National Bank of Microorganisms of the Institute of Investigations in Agricultural and Environmental Biosciences (INBA-CONICET) of the Agronomy School at University of Buenos Aires, Argentina.

### MALDI–TOF MS for bacterial identification

To identify NTM isolates, we used MALDI-TOF (Matrix Assisted Laser Desorption Ionization-Time of Flight) mass spectrometry [37]. Cultures were grown in TSA medium (Trypticase Soy Agar, Laboratorios Britania S.A), incubated at 37 ° C for 18h. Samples were processed on a Microflex MALDI-TOF MS spectrometer (Bruker Daltonics, Bremen, Germany) and analyzed using the coupled software FlexControl v3.0 (Bruker Daltonics). Protein # 1 standard (BTS, Bruker Daltonics) was included for calibration. All samples were analyzed in duplicate. The analysis was performed by direct extraction methodology "on spot". A colony was deposited without prior extraction step using a wooden stick and allowed to be air dried at room temperature on a metal plate in MALDI-TOF. The samples were fixed with 1 μl of formic acid and then with 1 μl of α-cyano-4-hydroxycinnamic acid to allow co-crystallization of the matrix solution with the sample at room temperature. The positrons were extruded linearly at an acceleration of 20 kV. The obtained spectra represent the sum of the ions obtained after the impact of 350 automatic shots of the laser. The spectra were analyzed in a range of m/z (mass/ionic charge ratio) of 3,500 to 20,000. Identification was performed using the MALDI BiotyperTM v3.1 program (Bruker Daltonics) by comparison of the mass spectra obtained for the microorganisms under study with those included in their database. A possible error of a +10 variation of the peak value of m/z was considered. The results were interpreted from the score assigned by the software to each sample (in the context of the analysis performed on the microorganisms under study). According to the criteria proposed by the manufacturer, a result was considered valid (accurate identification to the species level) whenever the score value was ≥2.0 [38].

### Phylogenetic analysis

Phylogenetic trees were constructed for all the identified bacteria, adding previously reported *Yokenella*, *Pantoea* and *Enteroccocus* 16S rRNA 1450 bp sequences. To evaluate the phylogenetic proximity of *Yokenella* sp isolates to those bacteria identified as *Klebsiella pneumoniae* in *N*. *viridula* guts collected in Brazil [24], we included sequences available from the type strain of *Yokenella regensurgei* ATCC 49455, other strain of *Yokenella* sp., the type strains *Klebsiella oxytoca* ATCC 13182, *Klebsiella michiganensis* ATCC BAA-2403 and *Klebsiella pneumoniae* ATCC 13885.

In a second tree, NvP01 sequence was compared with 23 type strains of *Pantoea* species. A third phylogenetic tree was constructed to analyze the proximity our enterococci to other *Enterococcus* sp type strains (*Enterococcus faecalis* JCM 5803 and *Enterococcus moriaviensis* ATCC BAA-383), and those enterococci identified in guts of *N*. *viridula* collected in Brazil [24].

All phylogenetic analyses were conducted in MEGA software version 6.06 [39]. Sequences corresponding to the 16S rRNA gene were aligned using the Muscle algorithm. A survey of genetic distances based on the alignments was performed and the Kimura 2-parameter [40] substitution model with Gamma corrections for variations of the mutation rate across sites was chosen. For gap treatment, complete deletions were considered. The neighbor joining algorithm [41] was used to generate the phylogenetic trees and they were validated with a bootstrap of 1000 replicates.

### Bacterial localization and cysteine protease activity in individual midgut ventricles of reared *N*. *viridula* adults

Stinkbug adults were kept under rearing conditions in plastic cages at 23°C; 10:12h dark/light; 60% humidity, and they were fed with mature rehydrated soybean seeds, dehulled sunflower seeds and unsalted peanuts seeds. Ten reared adults were dissected under sterile conditions and guts were microdissected and pooled to obtain samples of individual midgut ventricles. ARISA detection and plate count was performed as it is reported elsewhere. Cystein protease activity was measured according to Zavala et al (2008)[42] Here, cysteine proteinase activity was estimated by using the chromogenic substrate p-Glu-Phe-Leu-pNA. Then 10 μl of the 18× diluted enzyme was added to 20 μl of 0.38 mM p-Glu-Phe-Leu-pNA [in 0.1 M NaPhosphate, 0.3 M KCl, 0.1 mM EDTA, and 3 mM dithioerythreitol (pH 6.0)] and incubated at 37°C. Absorbance at 410 nm from wells on the microtiter plate was measured at 20-s intervals for 20 min with *N*. *viridula* guts enzymes. Initial rates of hydrolysis were estimated from the slopes of the resulting absorbance versus time graphs. One cysteine activity unit was defined as the amount of enzyme required to produce 1 mM 4-nitroaniline per minute at 37°C using p-Glu-Phe-Leu-pNA as a substrate under given assay conditions. Here, cysteine protease activity against specific substrate (p-Glu-Phe-Leu-NA; Sigma) is normalized with total protein content in the gut (Bradford-BioRad). Enzymatic kinetics curves were performed on a microplate spectrophotometer BIOTEK 808xl, with 402 nm filter. Enzymatic kinetics curves were performed on a microplate spectrophotometer BIOTEK 808xl, with 402 nm filter. The assay was performed in triplicate.

### Identification of V4 midgut symbiont in *N*. *viridula*

A consistent peak of 745 pb was detected in all field collected samples and in reared stinkbugs when bacterial community ARISA was performed (S2 Fig). Ventricle microdisecction allow us to locate this peak on V4 midgut ventricle and purified this ITS fragment from agarose gel using Agarose gel PCR purification kit. This fragment was cloned and sequence as it is reported in previous sections. After sequencing, BLAST data base was used to confirm bacterial origin and identify at family level.

### Bacterial gut community Automated Ribosomal Intergenic Spacer Analysis (ARISA)

To characterize stink bug gut bacterial community, ARISA electropherograms of total gut and isolated bacteria were compared. ARISA specifically amplifies 16S and 23S rRNA intergenic spacer and allows identifying the presence of uncultivable bacteria. The polymerase chain reaction (PCR) step was performed according to the method described by Kent and Bayne (2010)[43]. Intergenic spacers (ITS) were amplified with primers 23Sr (5´-GGGTTBCCCCATTCRG-3´) and 1406f (5´-TGYACACACCG CCCGT-3´) marked at the 5´end with 6-FAM fluorescent dye. Denaturing capillary electrophoresis was carried out for each PCR reaction using an ABI 3130 Genetic Analyzer (Applied Biosystems) at the Biotechnology Institute, INTA Castelar, Argentina. Estimation of DNA fragment sizes was accomplished by using synthetic molecular weight size standard ABI GeneScan^™^ 1200 LIZ. 748bp and 756bp peaks were used as positive control as they were consistently found in all samples (field collected and reared adults). As ARISA and bacterial count on agar plates were conducted in parallel, we were able to determine that sensibility of ARISA technique was at least of 10^3^ CFU/mg gut, as it was seen for Collecting event N° 25 (S3 Table).

### Functional digestive activities of NTM isolates

Glycolytic activity of enterobacteria and enterococci was followed by using the 50 carbon source fermentation strip API 50CHE (Biomerieux) accordingly to the manufacturer's recommendations. Fermentation of soybean seeds main components, sucrose, manose, cellulose and raffinose was recorded at 24 h and 48 h. Proteolytic activity was detected by plating (as a spot) 5 μl of overnight pure cultures adjusted to 0.1 McFarland onto Skim Milk Agar, incubated 96 h at 23-30-37 °C. Any transparent halo around the site of inoculation was considered as a positive proteolytic result. Lipolytic activity was evaluated by Rodhamine B-olive oil agar, incubated as described above, and any pink/orange fluorescent halo was considered as a positive result using a florescent lamp with an excitation wavelength of 350 nm [44].

### Soybean whole meal fermentation and cysteine protease inhibitory activity determination

Whole mature seeds of soybean cv. Williams were grounded with a coffee grinder to obtain fine flour. The flour was sieved through a 0.5 mm pore metal mesh and suspended 20% in sterile distilled water. Ten ml of the suspension were transferred into each of several 15 ml Falcon tubes. An aliquot was immediately stored at -20°C (non-pasteurized control). Remaining aliquots were pasteurized in a water bath at 50°C for 1 h to reduce protein denaturation, including cysteine protease inhibitors. 100 μl of a 10^8^ CFU/ml suspension of overnight cultures were inoculated in the pasteurized suspensions, and cultures were maintained for 24 h at 37 °C. One tube was left without inoculation of bacteria (non-inoculated control). After this period, soybean ferments and control were homogenized with vortex for 30 seconds and 1 ml aliquots of each ferment and control were centrifuged at 12000 g for 20 min to obtain cysteine protease inhibitors extracts. Cysteine proteases inhibitory activity of fermented and non-fermented (control) soybean whole meal extracts was measured against papain by following the release of *p*-nitroaniline (*p*NA; 37°C for up to 20 min at 410 nm) after adding the synthetic substrate *p*-Glu-Phe-Leu-pNA [42]. Briefly, 30 μl of 28 μg/ml papain was incubated in a 96-microplate with 0–10 μl of supernatant of soybean fermented extracts at 37°C for 10 min before addition of the substrate. Cysteine protease inhibitors concentration was normalized with total protein content in the gut (Bradford-BioRad)[42]. Enzymatic kinetics curves were performed on a microplate spectrophotometer BIOTEK 808xl, with 402 nm filter. The assay was performed in triplicate.

### Statistical analysis

To evaluate the relationship between the presence of bacteria in the gut of *N*. *viridula* and the host where stinkbugs were feeding on, Chi square (and Fisher´s exact) test was performed. To evaluate the efficiency of isolated bacteria to inactivate cysteine protease inhibitor of soybean whole meal, ANOVA with Dunnet posttest was performed, and non-inoculated pasteurized soybean meal was used as control. To evaluate the effects of incubation of bacteria with soybean meal on cysteine proteases inhibitory activity, a t-test was performed. All analysis were made with Prism 5.01 2007 (GraphPad Software Inc).

## Results

### Insect host survey

**Fig 1:**
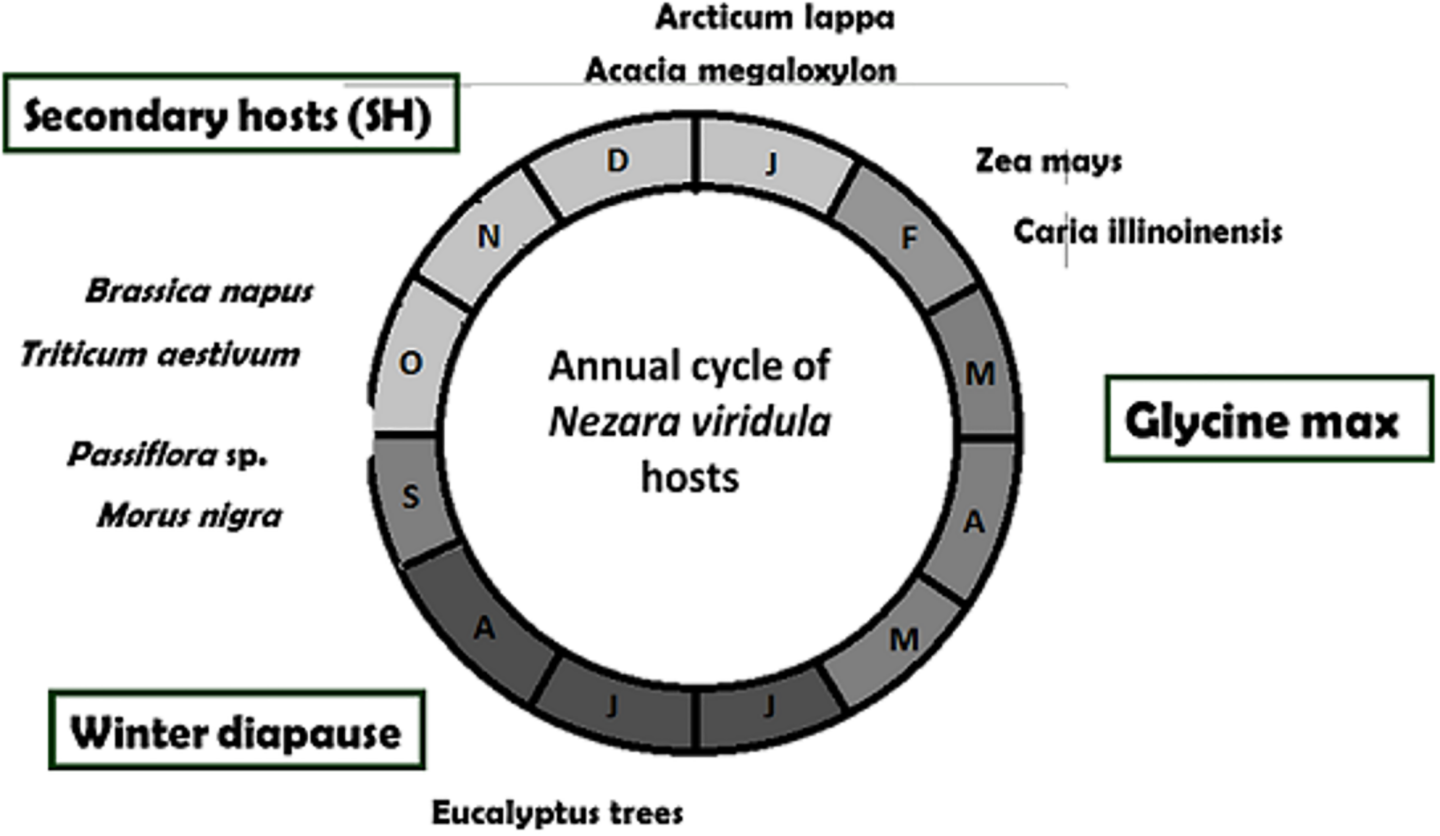
*Nezara viridula* hosts survey around the year cycle. Secondary hosts (SH) from late September to late January; soybean (primary host) from February to late May and under the bark of *Eucalyptus* trees (diapause) from June to late September.

Before we started the study of bacterial community in the gut of *N*. *viridula*, we performed a survey to determine the main hosts where these stinkbugs naturally feed on. During the southern hemisphere winter (June to August), *N*. *viridula* adults were found on diapause, sheltering under the bark of *Eucalyptus* trees present at the edges and in internal patches of soybean crops (Fig 1). In spring, as result of longer photoperiod and increase of average temperature, *N*. *viridula* begins to colonize different plant species as hosts (secondary hosts: SH) around the trees. From mid-September to late January, adults were found feeding on mulberry (*Morus nigra* L.), passion flower (*Passiflora* sp), honey locust (*Acacia megaloxylon*) and burdock (*Arcticum lappa*) (Fig 1). Stinkbugs were also collected from maize (*Zea mays*), rapeseed (*Brassica napus*), wheat (*Triticum aestivum*) and pecan (*Carya illinoinensis*) (Fig 1). We were not able to find representative number of stinkbugs between November and December, probably because of the low number of adults and the dispersion of stinkbugs among many plant species. However, from the beginning of February until the end of April stinkbugs moved from secondary hosts to soybean crop (primary host), where they were collected (Fig 1). *N*. *viridula* colonized soybean crops from the edges of the field during pods elongation (R4 according to Fehr and Caviness, [45] and the population started to grow inside the field. The last generation of stinkbugs turned to adult feeding on senescing soybean pods, thus they sought shelter under *Eucalyptus* bark to survive through winter (diapause) (Fig 1).

### Identification and characterization of midgut microbiota and its relationship with the insect host

Midgut microbial community analysis through ARISA and bacterial plate count of 173 stinkbugs from 26 collecting events revealed that *N*. *viridula* is associated with few species of bacteria (Table 1). Based on colony morphology, 21 gut bacteria were isolated and divided in two groups (Table 1). The first group, with a bacterial count over 10^4^ CFU/mg of gut, was identified as members of the *Enterobacteriaceae* and *Enterococcaceae* families (Non-Transient microbiota; NTM). These isolated bacteria were also detected by the culture independent technique ARISA. Sequence analysis of the genes coding for the 16S rRNA showed that seven isolates of *Enterobacteriaceae* were close to *Yokenella* (NvH01, NvO01, NvP02, NvR01, NvU01, NvU02, NvW01) with a 99% similarity (Table 1). These isolates were further confirmed as related to the *Yokenella* genus by MALDI-TOF MS bacterial identification. Isolates NvH01 and NvO01, were identified as *Y*. *regensburgei* with scores 2.04 and 2.07 respectively, while NvP02, NvW01, NvU01, NvU02 and NvR01, were identified as *Yokenella* sp. as their scores were <2 (1.73, 1.96, 1.94, 1.85 and 1.96, respectively) (S3 Table). In addition, we were able to identify one isolate as *Pantoea* sp (NvP01), and one as *Cedecea* sp (NvMJ01). Conversely, we found 4 gram-positive isolates to be *Enterococcus* sp (NvH02, NvM04, NvS01 and NvW02).

**Table 1:** Microscopic and molecular identification of 21 isolates from the midgut of field collected *N*. *viridula* adults.

Group	Strain*^1^	ARISA detection	log CFU/mg guton TSA*^2^	Gram stainig	16S ARNr V4 or 1492 bp*^3^	Selected for characterization*^4^
**Non -Transient microbiota**	NvH01	**Yes**	6	*Gram—bacilli*	*Yokenella* sp.	**Yes**
	NvO01	**Yes**	6	*Gram—bacilli*	*Yokenella* sp.	**Yes**
	NvP01	**Yes**	4	*Gram—bacilli*	*Pantoea* sp.	**Yes**
	NvP02	**Yes**	6	*Gram—bacilli*	*Yokenella* sp.	**Yes**
	NvR01	**Yes**	4	*Gram—bacilli*	*Yokenella* sp.	**Yes**
	NvU01	**Yes**	6	*Gram—bacilli*	*Yokenella* sp.	**Yes**
	NvU02	**Yes**	6	*Gram—bacilli*	*Yokenella* sp.	**Yes**
	NvW01	**Yes**	4	*Gram—bacilli*	*Yokenella* sp.	**Yes**
	NvMJ01	**Yes**	5	*Gram—bacilli*	*Cedecea* sp.	NO
	NvH02	**Yes**	7	*Gram+ rods*	*Enterococcus faecalis*	**Yes**
	NvS01	**Yes**	5	*Gram—bacilli*	*Enterococcus* sp.	**Yes**
	NvW02	**Yes**	5	*Gram+ rods*	*Enterococcus* sp.	**Yes**
	NvM04	**Yes**	7	*Gram+ rods*	*Enterococcus* sp.	**Yes**
**Transient microbiota**	NvI01	NO	1	*Gram+ bacilli*	*Streptomyces* sp.	NO
	NvI02	NO	1	*Gram + rods*	*MIcrococcus* sp.	NO
	NvJ01	NO	1	*Gram+ rods*	*Staphylococcus* sp.	NO
	NvJ02	NO	1	*Gram + bacilli*	*Bacillus* sp.	NO
	NvM02	NO	<1	*Gram + bacilli*	*Bacillus* sp.	NO
	NvM01	NO	<1	*Gram—bacilli*	*Bacillus* sp.	NO
	NvO02	NO	1	*Gram + bacilli*	*Bacillus* sp.	NO
	NvS02	NO	<1	*Gram+ rods*	*Micrococcus* sp.	NO

*^1^: Isolate identification code: NvXN°; Nv (*N*. *viridula)* X (collecting event) N° (isolate number).

*^2^: bacterial count was performed on Trypticase Soy Agar, overnight, 37°C.

*^3^: V4 sequences were compared against GenBank database. Complete sequence was compared against SILVA databe.

*^4^: ARISA detected isolates were chosen for further characterization.

Among isolates with sporadic presence and counts lower than 100 CFU/mg of gut, that were not detected by ARISA (Transient microbiota; TM), other different bacteria were identified, such as *Bacillus* sp. (NvJ01, NvM02 and NvO02), *Streptomyces* sp. (NvI01), *Micrococcus* sp. (NvI02 and NvS02) and *Staphylococcus* sp. (NvJ01) (Table 1). Since strict aseptic conditions were used during dissection and plating, external contamination was preliminarily discarded. ARISA profiles of identified bacteria allowed evaluating gut insect samples without further specific isolation. For example, *Yokenella* sp. consistently showed ARISA peaks of 626, 710, 780 bp; *Pantoea* sp. peaks of 651, 667 and 858 bp; *Cedecea* peaks of 659 and 815 bp and *Enterococcus* sp. peaks of 502 and 602 bp (S2 Table).

**Fig 2:**
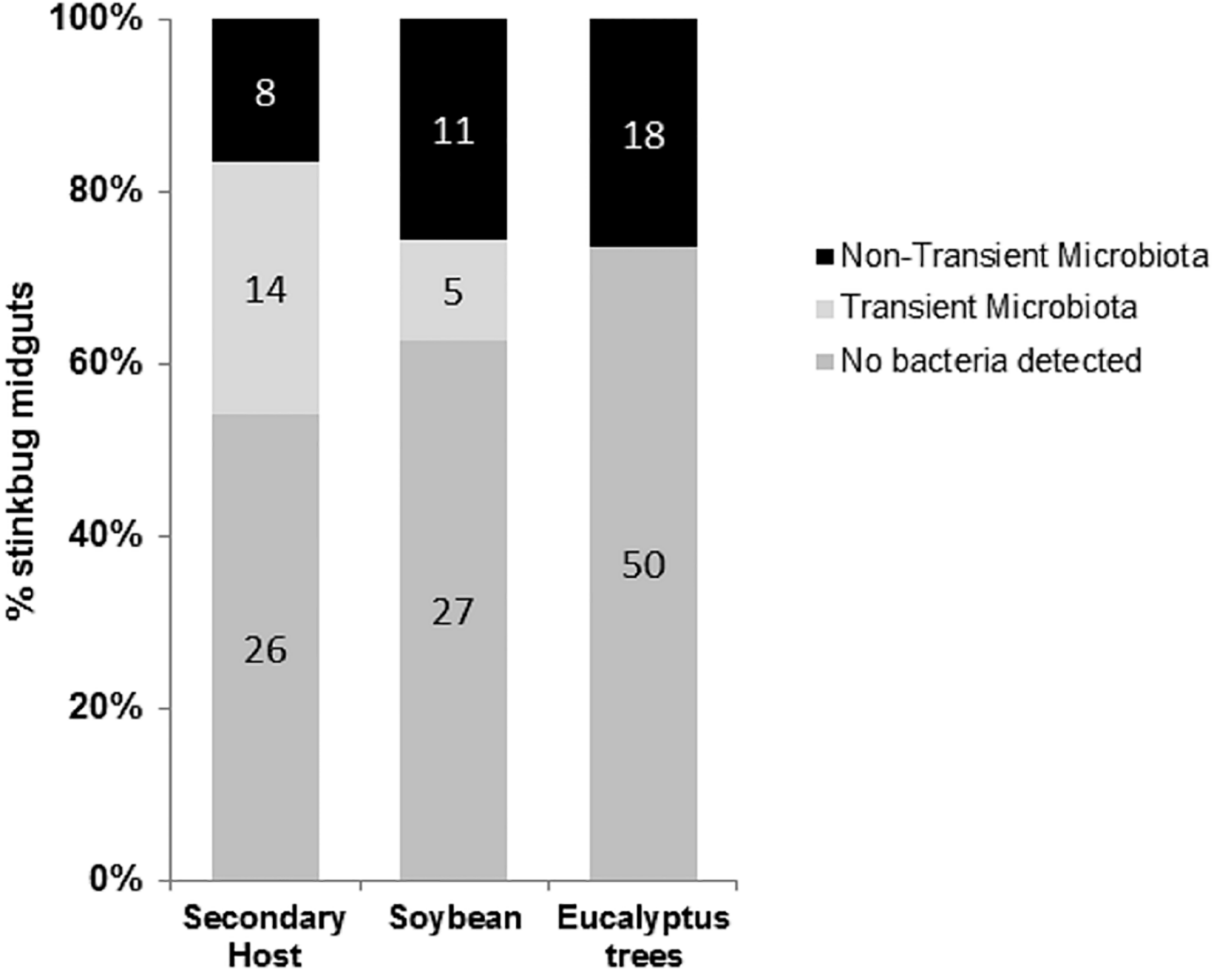
Presence/absence of gut microbiota in *N*. *viridula* adults associated with the insect host. Presence of non-transient (black) and transient (light grey) microbiota or absence of bacteria (Not infected; dark grey) in *N*. *viridula* adult’s V1-V3 midgut ventricles; and its distribution related to insect host: secondary hosts (SH), soybean and under the bark of *Eucalyptus* trees. Numbers correspond to insect gut dissected.

We found a strong association between gut bacterial community and plant host of *N*. *viridula* (Fisher´s test—X^2^: 23.0; df: 4, p = 0.001; Fig 2). NTM was found in the guts of 17% of stinkbug adults feeding on SH and in 26% of those feeding on soybean, and in 26.5% of stinkbugs in diapause under the bark of *Eucalyptus* trees (Fig 2). While transient microbiota was present in 29% of stinkbugs guts that were feeding on SH and in 12% of those feeding on soybean, this microbiota was absent in stinkbugs during diapause (Fig 2). Also, 54%, 62% and 73.5% of stinkbugs guts were found to be free of cultivable bacteria on TSA or ARISA detectable bacteria, when feeding on secondary hosts, soybean or under the bark of *Eucalyptus* trees, respectively.

Gut bacterial community richness of collected samples (α diversity) ranged between cero and five, and was restricted to three main phyla: *Enterobacteriaceae*, *Enterococcaceae*, *Bacillaceae* (S2 Table). Regarding enterobacterial isolates, *Yokenella* sp. was detected in ten collecting events (two from SH; three from soybean, and five from *Eucalyptus* trees) (S2 Table), while *Pantoea* sp. was detected in two collecting events (sampled in soybean and in *Eucalyptus* trees) and *Cedecea* only in one (sampled in soybean) (S2 Table). Enterococci were isolated from five collecting events (two sampled in SH, one in soybean and two in *Eucalyptus* trees). *Enterococcus* sp. and *Yokenella* sp. were found cohabiting *Nezara*´s gut (collecting events N° 1, 9 and 23), where *Enterococcus* bacterial count was always one logaritmic order over *Yokenella* (S2 Table). *Yokenella* was more abundant (10^6^ CFU/mg gut) when inhabiting the midgut without *Enterococcus* competition (collecting events N° 2, 10, 14, 17, 19; S2 Table).

**Fig 3:**
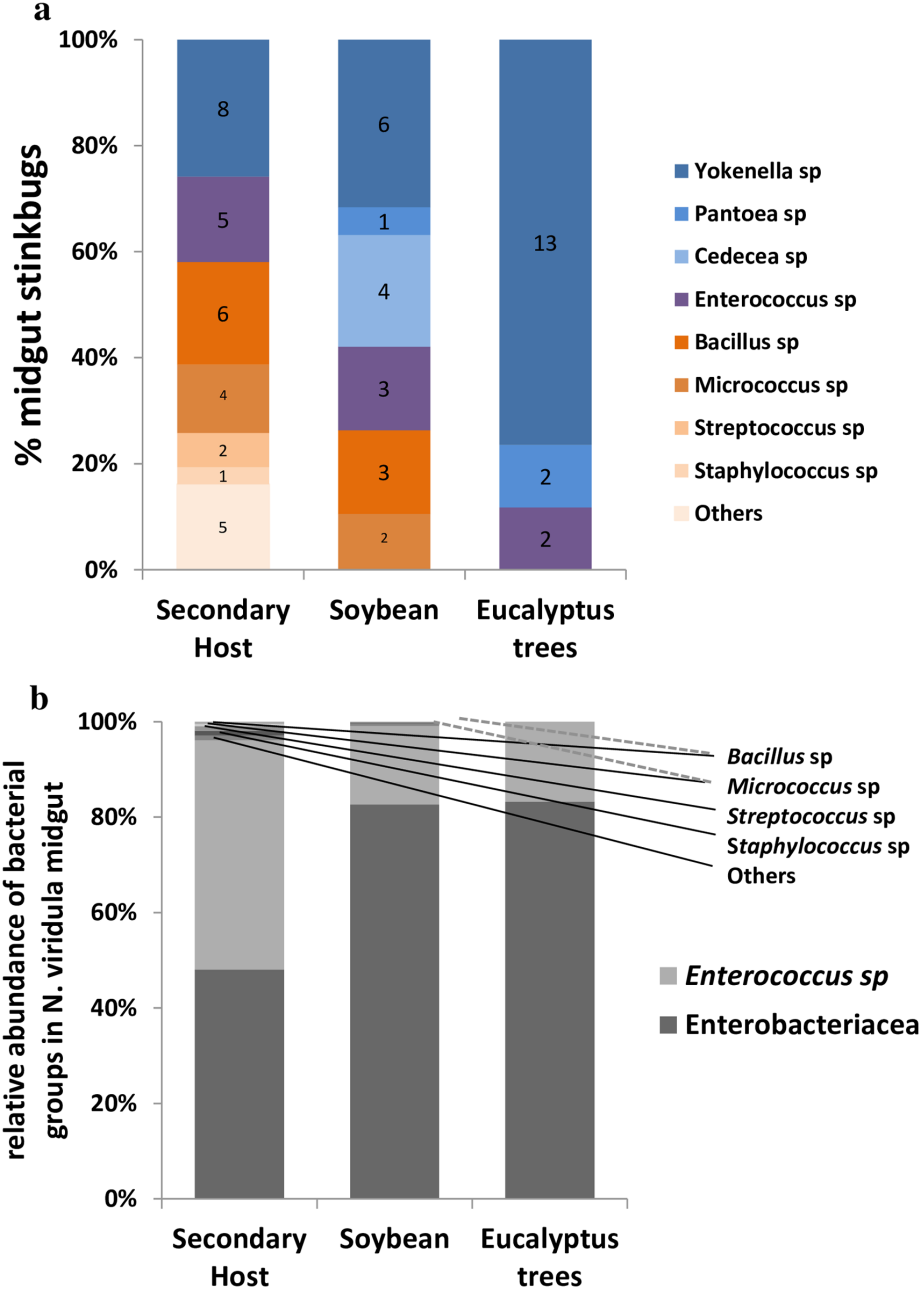
Gut bacterial communities associated with the insect host. Bacteria species inhabiting *Nezara viridula* infected V1-V3 midgut ventricles and its distribution among insect hosts (a) and relative abundance (CFU/mg gut x n° insects positive ^-1^) of bacterial groups of infected *N*. *viridula* V1-V3 midgut ventricles and its distribution among hosts (b). *Enterobacteriaceae groups *Yokenella*, *Cedecea* and *Pantoea* species. Numbers correspond to insect gut dissected.

*Yokenella* was the most frequent NTM in collected stinkbugs. We found *Yokenella* in eight stinkbugs feeding on SH and in six feeding on soybean, and in 13 of those found in *Eucalyptus* trees (Fig 3a). Conversely, *Bacillus* sp. was the most frequent among bacteria of the transient microbiota group. We found *Bacillus* sp. in six stinkbugs feeding on SH and in three feeding on soybean crop (Fig 3a). Relative abundance analysis of NTM showed that *Enterobacteriaceae* represented 47% of bacterial communities in stinkbugs feeding on SH, and 80% in stinkbugs feeding on soybean, and in those found under the bark of *Eucalyptus* trees (Fig 3b). Moreover, *Enterococcus* sp. was 47% of the bacterial community in stinkbugs feeding on SH and 19% in those feeding on soybean, and 20% in the stinkbugs collected from *Eucalyptus* trees (Fig 3b). Finally, transient microbiota did not exceed 5% of bacterial population in the gut of stinkbug feeding on SH and reached 1% in stinkbug feeding on soybean, and was not detected in those in diapause (Fig 3b).

### Bacterial localization in the midgut of *N*. *viridula*

Adults of *N*. *viridula* reared in laboratory were microdissected to evaluate digestive activity and bacterial communities of individual ventricles (V). ARISA of midgut dissected ventricles V1-V4 of *N*. *viridula* revealed the presence of enterococci and enterobacteria in ventricles V1-V3, while caeca (V4) harbored a non-cultivable bacterium with 748 and 756 bp ITS fragments (S2 Fig). Sequencing of the 748 bp fragment confirmed bacterial origin related to the *Enterobacteriaceae* family. Cysteine protease activity was higher in ventricles V2-V3 (9.0 ± 1.5 activity units and 16.8 ± 2.2 activity units, respectively) than in V4 (0.5 ± 0.05 activity units), indicating that ventricles V2-V3 are specialized for diet degradation and absorption (S2 Fig).

### Phylogenetic analysis of *Enterobacteriaceae* and *Enterococcus* sp.

**Fig 4:**
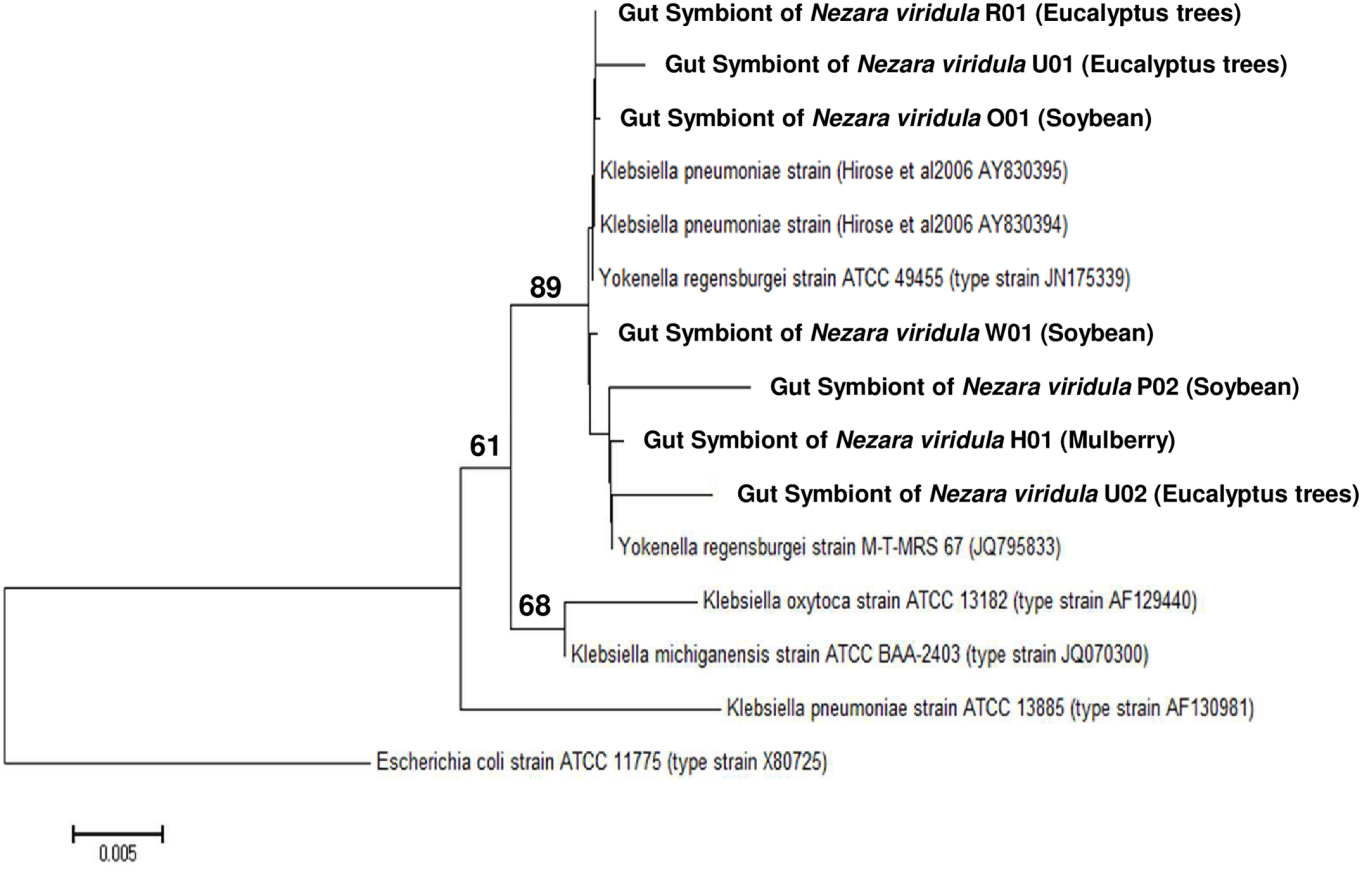
Phylogenetic placements of *Yokenella* strains (bold letters). We constructed a tree that included our isolates, those described as *K*. *pneumoniae* by Hirose et al. (2006), the species reference strains *K*. *pneumoniae* ATCC 13885, *K*. *michiganensis* ATCC BAA-2403, *K*. *oxytoca* ATCC 13182, *Y*. *regensburgei* ATCC 49455 and one additional strain of *Y*. *regensburgei*. Gene bank accession numbers and insect host of bacteria isolated in this study appear in parentheses *Escherichia coli* ATCC 11775 was included as an outgroup to root the tree. *K*. *michiganensis* and *K*. *oxytoca* were included because a blast search in the RefSeq database indicates that both these species and *Y*. *regensburgei* lie at closer genetic distance to the Hirose´s strains than *K*. *pneumoniae*. Only the bootstrap values greater than 60% are shown. Scale bar states for the phylogenetic distance with a common ancestor.

16S rRNA sequences of gut isolates from adults of *N*. *viridula* matched with *Yokenella* sp. sequences, which is a species phylogenetically close to *Klebsiella* (Fig 4). The phylogenetic analysis of the 16S rRNA gene sequences revealed two different clusters (89% bootstrap confidence); one of the (NvR01, NvO01 and NvU01) match showed a high similarity to the type strain *Yokenella regensburgei* ATCC 49455, while the other (NvH01, NvP02, NvU02 y NvW01) matched closer to a different strain deposited as *Yokenella* (S3 Table). The first group of *Yokenella* sp. (NvR01, NvO01 and NvU01) also matches closer to *Klebsiella pneumoniae* strains reported by Hirose (24), which are clearly separated from other *Klebsiella* species, suggesting that the bacteria isolated in Brazil are more likely to be *Yokenella* than *Klebsiella*.

**Fig 5:**
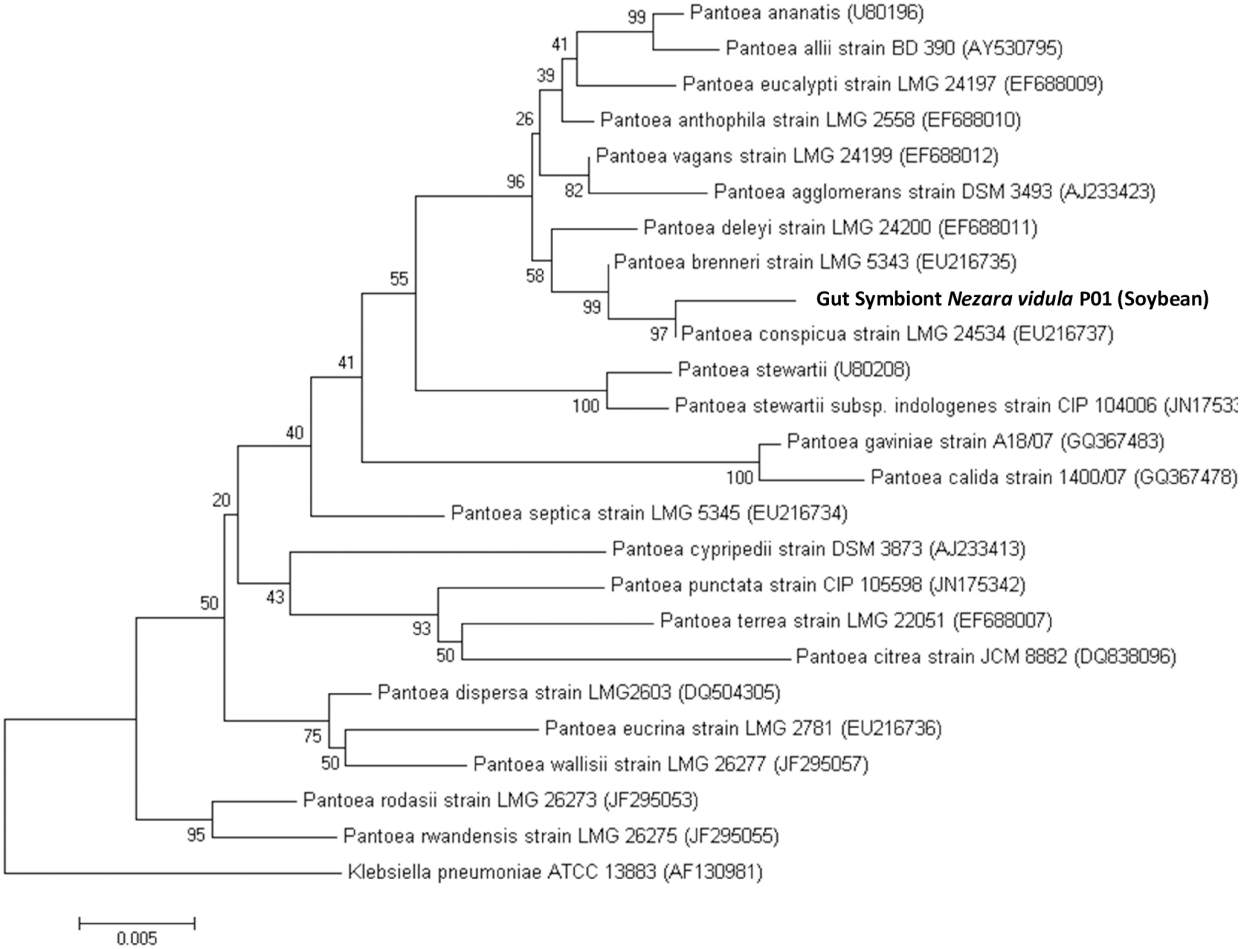
Phylogenetic positioning of *Pantoea* NvP01 (bold letters). We constructed a tree that included our isolate and type strains of 22 species of *Pantoea* and a subspecie of *Pantoea stewartii*. Gene bank accession numbers and insect host of bacteria isolated in this study appear in parentheses. The sequence of the type strain *Klebsiella pneumoniae* ATCC 13883 was included as an outgroup to root the tree. Only the bootstrap values greater than 65% are shown. Scale bar states for the phylogenetic distance with a common ancestor.

Another enterobacteria was identified as *Pantoea* sp., (NvP01) with a closer match to *P*. *conspicua*. Phylogenetic analysis included 22 species of *Pantoea* and one subspecies of *P*. *stewartii* (Fig 5). *Pantoea* sp. was also detected with ARISA from stinkbugs collected on *Eucalyptus* trees as it presented the same peak pattern as *Pantoea* sp. NvP01.

**Fig 6:**
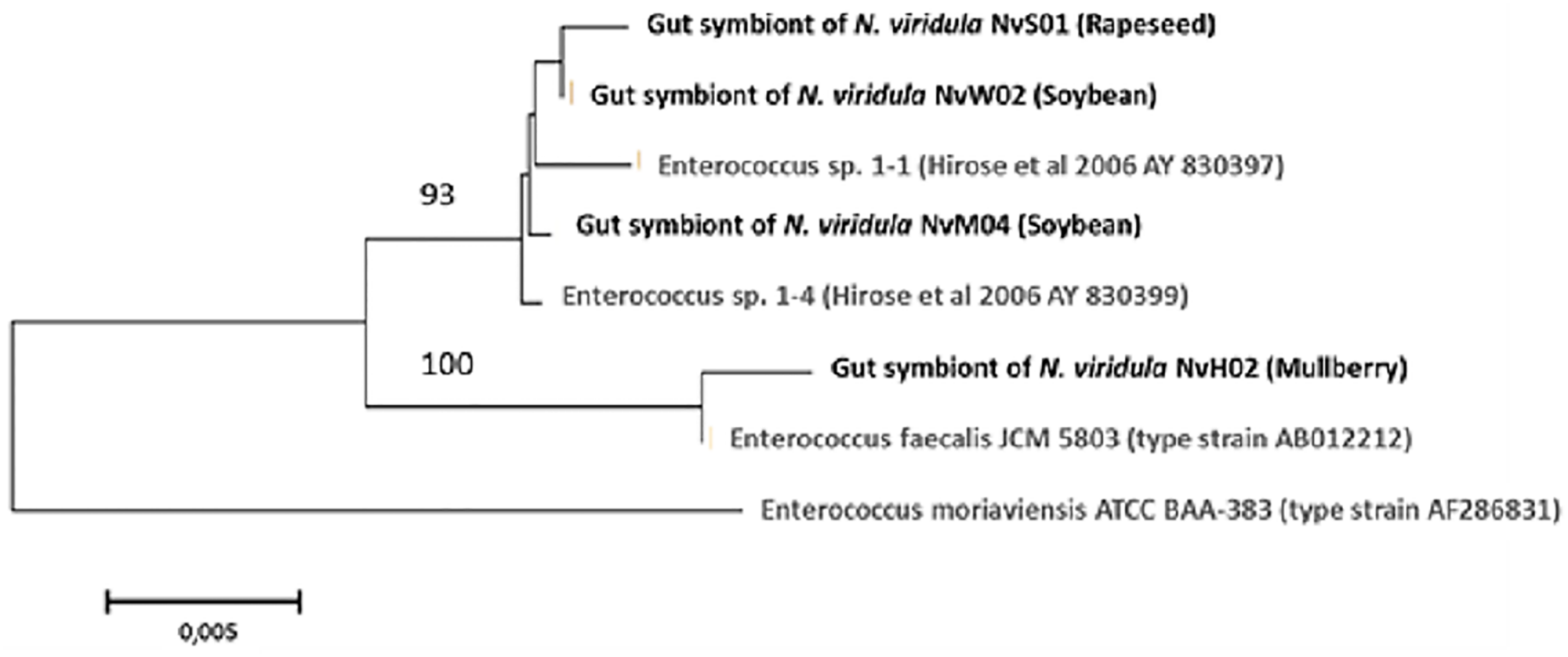
Phylogenetic positioning of *Enterococcus* isolated strains (bold letters). We constructed a tree that included our isolates, those described as *Enterococcus faecalis* by Hirose et al. (2006), and type strain *Enterococcus faecalis* JCM 5803. Gene bank accession numbers and insect host of bacteria isolated in this study appear in parentheses. The sequence of the type strain *Enterococcus moraviensis* ATCC BAA-383 was included as an outgroup to root the tree. Only the bootstrap values greater than 65% are shown. Scale bar states for the phylogenetic distance with a common ancestor.

We also isolated and identified an *Enterococcus* sp. strain (NvH02), very closely related (overall genetic distance 0.002 and similarity greater than 99%) to the *Enterococcus faecalis* type strain (Fig 6 and S4 Table). In a separated branch, our other *Enterococcus* sp. isolates (NvM04, NvS01 and NvW02) grouped together with those identified in *N*. *viridula* in Brazil [24] (Fig 6 and S4 Table). These bacteria had also a 99% similarity to *E*. *faecalis*, and the search in the RefSeq database of NCBI confirmed that the closest member was indeed *E*. *faecalis*.

### In vitro enzymatic activities of bacteria in ventricles V1-V3 of *N*. *viridula*

To test whether gut isolated bacteria can help stinkbugs to digest soybean, *in vitro* analysis of enzymatic activities using a specific culture media were performed. None of the *Yokenella* isolates were able to utilize sucrose, the main sugar in soybean, and had no proteolytic activity on casein under aerobic or fermentative conditions (Table 2). *Yokenella* sp. NvH01 obtained from stinkbugs feeding on *Morus nigra* and *Yokenella* sp NvU01 and NvW01 isolated from those collected on *Eucalyptus* trees, had cellulase, maltase, esculinase and rafinase activity, evidencing α 1–2, β 1–4, α 1–4, β 1–6, α 1–6 glycosidase activity, respectively (Table 2). In addition, *Yokenella* sp. NvH01, *Yokenella* sp. NvO01 isolated from insects feeding on soybean and *Yokenella* sp. NvR01 and NvU02 isolated from insects in diapause, were positive for lipase activity, as they showed pink fluorescent colony and degradation halos on Rhodamine B/Olive oil Agar plates (Table 2). *Pantoea* sp. NvP01 isolated from insects feeding on soybean was able to degrade sucrose, cellulose and maltose, but was not able to perform any proteolytic or lipolytic activity (Table 2).

**Table 2:** Enzymatic activities of isolated strains with significance in soybean digestion.

	*Enzymatic activity*	Glycolytic*^1^					Proteolytic*^2^		Lipolytic*^3^
		*α 1–2 glucoside*	*β 1–4 glucoside*	*α 1–4 glucoside*	*β 1–6 glucoside*	*α 1–6 galactoside*	*Total Proteases*	*Cystein proteases*	*lipases*
	*Substrate*	Sacarose	Cellulose	Maltose	Esculine	Rafinose	Casein	P Glu Phe Leu NA	Olive oil
***Yokenella* sp**.	**NvH01**	**-**	**+**	**+**	**+**	**+**	**-**	**-**	**+**
	**NvU01**	**-**	**+**	**+**	**+**	**+**	**-**	**-**	**-**
	**NvW01**	**-**	**+**	**+**	**+**	**+**	**-**	**-**	**-**
	**NvO01**	**-**	**-**	**-**	**-**	**-**	**-**	**-**	**+**
	**NvU02**	**-**	**-**	**-**	**-**	**-**	**-**	**-**	**+**
	**NvR01**	**-**	**-**	**-**	**-**	**-**	**-**	**-**	**+**
	**NvP02**	**-**	**-**	**-**	**-**	**-**	**-**	**-**	**-**
***Pantoea* sp**.	**NvP01**	**+**	**+**	**+**	**-**	**-**	**-**	**-**	**-**
***Enterococcus* sp**.	**NvH02**	**+**	**+**	**+**	**+**	**-**	**-**	**-**	**-**
	**NvS01**	**+**	**+**	**-**	**+**	**-**	**-**	**-**	**-**
	**NvM04**	**+**	**+**	**-**	**+**	**-**	**-**	**-**	**-**
	**NvW02**	**+**	**+**	**-**	**+**	**-**	**-**	**-**	**-**

*^1^. Bacterial glycolytic activities were evidence trough API 50CH strips (Biomerieux).

*^2^: Bacterial proteolytic activity were evidence on Skim Milk Agar and broth, and against specific cysteine protease substrate GluPheLeu—pNA (Sigma).

*^3^: Bacterial Lipolytic activity was evidence on Rhodamine B-Olive oil- Agar.

All of the enterococci strains isolated from insect feeding on *Morus nigra* (NvH02), rapeseed (NvS01), soybean (NvM04) or from stinkbugs in *Eucalyptus* trees (NvW02), were positive for utilization of sucrose, cellulose and esculine, and negative for utilization of raffinose (Table 2). *E*. *faecalis* NvH02 was also positive for utilization of maltose. No isolated enterococci strain showed proteolytic or lipolytic activities under the conditions of the assay.

### Enterobacteria and enterococci isolated strains decrease protease inhibitor activity of soybean flour during fermentation

**Fig 7:**
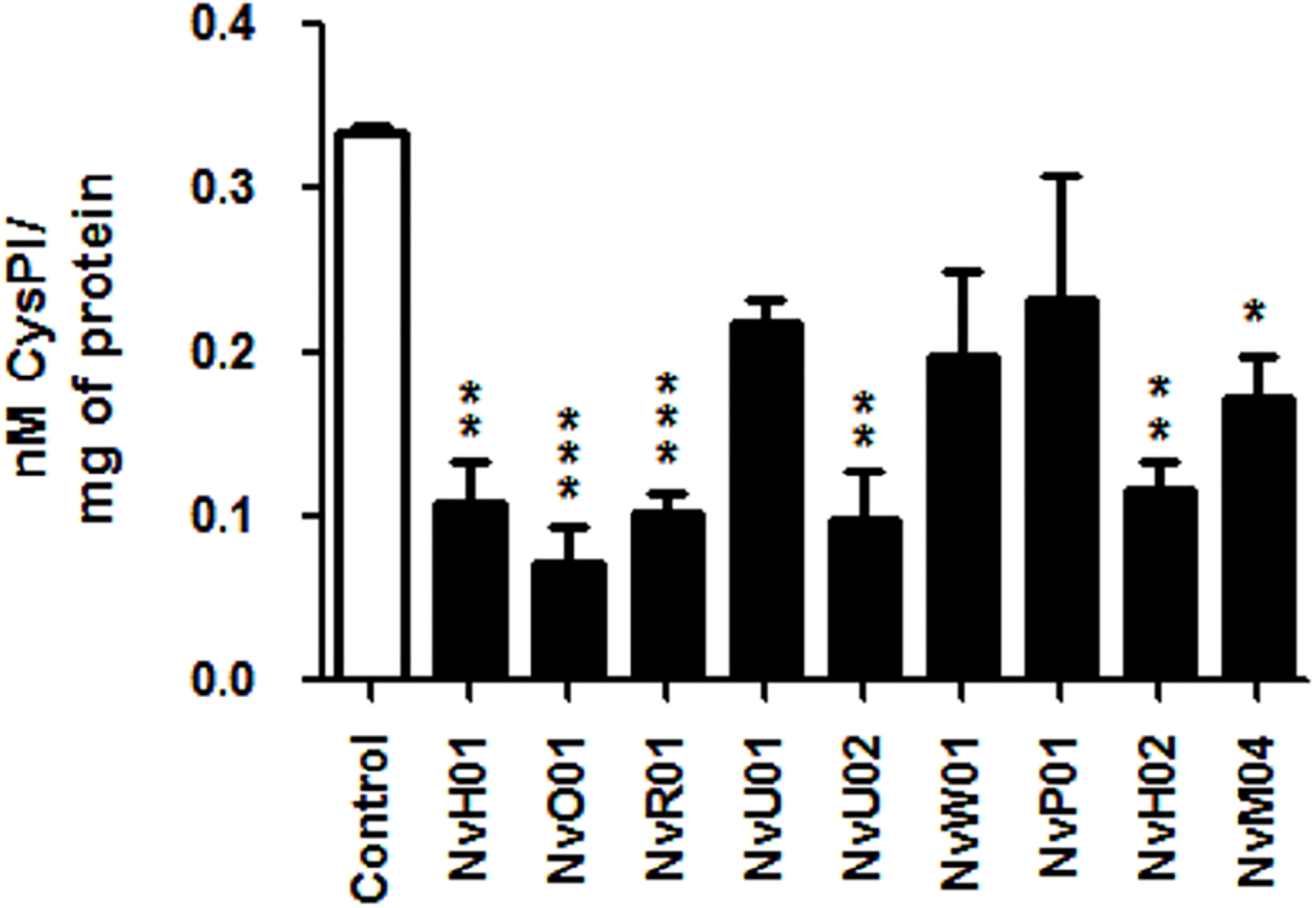
Inhibition capacity of fermented soybean cv. Williams whole meal extracts against cysteine protease papain. Whole meal was inoculated with non-transient microbiota (NTM) isolated bacteria and fermented during 24h at 37°C. *Yokenella* sp. NvH01, NvO01, NvR01, NvU01, NvU02 and NvW01; *Pantoea* sp. NvP01; *Enterococcus faecalis* NvH02 and *Enterococcus* p: NvM04. Control extracts correspond to a pasteurized non-inoculated and incubated soybean whole meal suspension.

To determine whether soybean protease inhibitors can be deactivated through fermentative process by bacteria from the midgut of stinkbugs, soybean flour was inoculated with representatives of the different isolated microorganisms and incubated. Incubation of the control (non-inoculated soybean flour) increased the inhibitory capacity of papain activity by 21.3%, probably due to hydration of soybean meal and activation of cysteine protease inhibitors (p = 0.04). However, the fermentative activities of *Yokenella* sp. NvH01, NvO01, NvR01 and NvU02, and *Enterococcus faecalis* NvH02 and NvM04 reduced the inhibitory capacity of soybean cysteine protease inhibitors from 35% to 75% (p<0.001; Fig 7). There were no significant differences among the different tested microorganisms (ANOVA; p = 0.07; Fig 7). Finally, bacterial cysteine proteases activity on fermented soybean meal was evaluated to identify potential activity that could mask papain inhibition. There was no cysteine proteases activity detected for any isolated strain or control.

## Discussion

Changing gut environment by bacterial communities allow insects to rapidly adapt to new hosts and to tolerate plant chemical defenses [10,16,20,23,46–49]. To test the hypothesis that midgut bacterial community of stinkbugs (*N*. *viridula*) deactivates chemical defenses of soybean developing seeds, we identified and characterized midgut microbiota of stinkbugs collected from soybean crops, different secondary plant hosts (SH) or *Eucalyptus* trees (Fig 1). Our study demonstrated that while more than 54% of *N*. *viridula* adults collected in the field had no detectable bacteria in V1-V3 midgut ventricles, the guts of the rest of stinkbugs were colonized by non-transient microbiota (NTM) and transient microbiota not present in stinkbugs at diapause (Fig 2).

Whereas transient microbiota had low abundance and was represented by a variable number of genera, such as *Bacillus* and *Micrococcus*, NTM microbiota was composed by enterobacteria and enterococci, which were represented by *Yokenella* sp., *Pantoea* sp., *Cedecea* sp. and *Enterococcus* sp. isolates (Table 1, Fig 3 and S2 Table). ARISA together with MALDI-TOF and 16S rRNA sequencing techniques permitted us identify the NTM (S3 Table), and phylogenetic trees allowed us positioning *Enterococcus* sp. *Yokenella* sp. and *Pantoea* sp. among similar species, and even suggesting that some microorganisms previously isolated were perhaps erroneously identified (Figs 4–6). Our *in vitro* results suggests that stinkbugs NTM may impact on nutrition, detoxification and deactivation of chemical defenses (Fig 7 and Table 2), indicating that *Enterococcus* sp. *Yokenella* sp. and *Pantoea* sp. isolated strains might help stinkbugs to feed on soybean developing seeds in spite of its chemical defenses. To our knowledge no study before has characterized the biological functions of *N*. *viridula* microbiota.

Stinkbugs collected from different areas showed that NTM was present in midgut ventricles V1-V3 where the highest enzymatic activity levels were measured, and suggested that *Yokenella* sp., *Pantoea* sp. and *Enterococcus* sp. may play an important role on insect nutrition and deactivation of chemical defenses (Fig 3). Midgut isolates of NTM were able to degrade galactosyl derivatives of sucrose, such as raffinose (Table 2), which is the second more abundant sugar in soybean seeds and is considered responsible of reducing digestibility because it has some activity as protease inhibitor [50]. Moreover, digestion of raffinose results on lower pH and prebiotic short chain fatty acids [51]. In addition, gut bacteria may deactivate protease inhibitors (Fig 7), which are the main defense of soybean against insect herbivores that can decrease the activity of digestive cysteine proteases of stinkbugs, reducing insect performance [1,52]. Although biochemical characterization of *Enterococus* sp. *Yokenella* sp. and *Pantoea* sp. isolated strains showed no extracellular proteolytic activity (Table 2), the NTM reduced the inhibitory capacity of cysteine protease activity of soybean whole meal after 24h of *in vitro* fermentation (Fig 7), and might help stinkbugs to feed on soybean developing seeds. Furthermore, midgut isolates of NTM showed β-glycosidase and α-galactosidase activities, and might hydrolyze the glyosidic bond of the isoflavonoids, genistin and daizin in the insect gut (Table 2). Isoflavonoids participate in the defense against insect attack, and damage produced by *N*. *viridula* increases these phenolic compounds production in attacked seeds [1,2,53,54]. It is not clear yet whether the isoflavonoids glycosides or aglycones are toxic to stinkbugs. Although NTM may play a role in helping stinkbugs to feed on soybean, we did not detect any bacteria in V1-V3 ventricles in more than 54% of collected insect (Fig 2).

Microorganisms of plant ecto- and endophytic communities are ingested during insect feeding and some of them can become part of the gut microbiota, depending on pH, enzymatic activity, redox potential and other intestinal conditions [8,9,55]. Midguts of *N*. *viridula* adults are acidic with a pH of 4.5–6.5, with sectored compartments and high enzymatic activity [24,33]. The lack of bacteria or low diversity of NTM found in V1-V3 of many stinkbugs (Figs 2 and 3), may be explained by feeding behavior, moulting, gut biochemical and physiological characteristics, non-gregarious behavior, among others [8,9,55,56]. Non gregarious behavior of *N*. *viridula* adults limit oral-fecal transference of bacteria and conformation of complex gut bacterial communities, as can be seen in social insects, which allows the stability of symbiotic bacteria in the population [8,9,55,56]. In addition, piercing sucking feeding behavior of *N*. *viridula* could limit horizontal transfer of bacteria by ingestion of endophytes, leaving behind phyloplane bacteria [10,57].

Host also can exert control over their microbiota in midguts by monitoring and targeting the species to either promote or hinder their proliferation [58]. We found that only few species of bacteria (NTM) can reside in the midgut of *N*. *viridula* (Table 1). Detecting harmful and beneficial traits of bacteria is a robust way for a host to monitor microbiota. There is a significant variability in antimicrobial peptides in host species, suggesting that the secretion of theses peptides from the host epithelium helps to determine which microbial genotypes prosper [59]. In addition, host diet has a major impact on the available resources within insects, which results in the microbial species dominating in the midgut. The diversity and frequency of NTM were higher in stinkbugs feeding on soybean than in those feeding on secondary hosts (Figs 2 and 3). This may be due to diet quality as soybean is a better substrate for enterobacteria [60]. Also, these bacteria had the ability to remain in the intestine during diapause (*Eucalyptus* trees) probably by colonization of midgut epithelium or the lumen (Fig 3). During isolation of NTM microbiota, enterobacteria showed fast *in vitro* growing (data not shown), suggesting a fast lumen colonization.

To compare the phylogenetic relations of the NTM species isolated from V1-V3 of stinkbugs collected from the field, we used type strains of the related species to perform 16S rRNA alignment trees, which has been the primary reference for bacterial phylogeny [61]. We found close phylogenetic relationship between bacteria identified in this study (Figs 4 and 5) with those previously isolated from midguts of *N*. *viridula* in Brazil [24]. Some of our isolates are closely related to others previously identified as *K*. *pneumoniae*, [24] our identification (including MALDI-TOF identification) suggest that the isolated bacteria are *Yokenella* sp. rather than *K*. *pneumoniae* (Fig 4). Some biochemical tests also would discard them as klebsiellas, but we do not have access to the microorganisms previously isolated. Different *Enterococcus* sp identified in our study are also closely related to those identified in Brazil (Fig 6). However, our discrimination among the different enterococci may not be sensitive enough. It is possible that *Yokenella* sp. and *Enterococcus* sp. might have been associated to *N*. *viridula* probably during migration from equatorial regions of Brazil to southern fields in Argentina or through a close interaction with soybean, explaining the presence of the bacterium species in the gut of stink bugs. *Yokenella* sp. has also been associated to the firebug (*Pyrrhocoris apterus*; Hemiptera, Pyrrhocoridae), a common cotton pest in Europe [62]. In addition, we isolated and identified *Pantoea* sp. from guts of stinkbugs collected from two different sites (Fig 5 and Table 1), which to our knowledge has never been identified in stinkbugs before. This bacteria has been previously isolated from different environmental samples (e.g., water, soil, plant material) and insects, including mosquitoes (Diptera), thrips (Thysanoptera), bees (Hymenoptera), and hemipterans [55], suggesting that this gram negative rod has a wide ecological distribution.

Our study showed that *N*. *viridula* adults feed on many different hosts, and suggested a strong association between gut bacterial community and plant hosts (Fig 2). Field surveys showed that adults of *N*. *viridula* feed on soybean crops, in winter move to *Eucalyptus* trees to spend diapause, and then in spring start to feed on different SH such as, mulberry (*Morus nigra* L.), passion flower (*Passiflora* sp.), honey locust (Acacia megaloxylon) and some crops like, maize (*Zea mays*), rapeseed (*Brassica napus*), wheat (*Triticum aestivum*) (Fig 1). *Eucalyptus* trees unable the insect to adapt to non-tropical regions, as they are used for sheltering during cold winters [6,63,64]. Although secondary hosts are not suitable for correct nymph development, they allow adults emerging from diapause to obtain resources to begin a new cycle [65–67]. Since between 17% and 26% of stinkbugs collected in the field from all hosts contain NTM in the midgut (Fig 2), NTM may help *N*. *viridula* to tolerate plant defenses and feed on different hosts. Enterobacteria and enterococci that infected the midgut of *N*. *viridula* may change the biochemical environment of guts, improving digestibility trough inactivation of protease inhibitors and other antinutrients. However, the small number of stinkbugs infected with NTM could indicate possible detrimental effects of these bacteria over the insect. Future work will focus on the effects of *Enterobacteriaceae* and *Enterococcus* midgut colonization on *N*. *viridula*.

## Supporting information

S1 Table Geographical placement of collecting sites. (PDF)

S2 Table Bacterial communities in the midgut of *Nezara viridula* associated to the insects hosts. (PDF)

S3 Table Bacteria isolated in this work and those used to build phylogenetic trees of Yokenella. (PDF)

S4 Table Bacteria isolated in this work and those used to build phylogenetic trees of Enterococcus sp. (PDF)

S1 Fig Map of Argentina (a) and a zoom of central east Argentina (b) were 26 collecting events were performed during 2012–2014. *Nezara viridula* adults were handpicked from secondary hosts (light grey spots), Soybean (dark grey spots) or from under de bark of Eucalyptus trees (black spots).
(PDF)

S2 Fig **(a) Cysteine protease activity of *N*. *viridula* V1-V4 midgut ventricles. Statistical differences are denoted by different letters. (b) Distribution of ARISA detected bacteria among *N*. *viridula* V1-V4 midgut ventricles**. Bacterial ITS fragments appear as blue peaks and LIZ 1200 weight standard fragments appear as yellow peaks. On a black square are 748 y 756bp cloacae symbiont ITS fragments. Numbers are reference for weight standard.
(PDF)
