# Supplementary material for: Characterized non-transient microbiota from stinkbug (Nezara viridula) midgut deactivates soybean chemical defenses
Source: PLoS One. 2018 Jul 12;13(7):e0200161. doi: 10.1371/journal.pone.0200161 (PMC6042706; doi:10.1371/journal.pone.0200161)
Supplement: S1 Table — (PDF) [file pone.0200161.s001.pdf]

S1 Table. Geographical placement of collecting sites.

Site Nº	Locality	Province	Latitude	Longitude	Collecting event reference*		
					SH	Soybean	Eucalyptus
1	Carabelas	Buenos Aires	-34,037867	-60,871811	H, A	M	U; R
2	Lujan	Buenos Aires	-34,569906	-59,118805	I, J	T	
3	La Plata	Buenos Aires	-35,014814	-58,069611	PC		
4	Pila	Buenos Aires	-35,980229	-57,994852		O, P	PA, PL
5	Marcos J	Córdoba	-32,699489	-62,100220		MJ	
6	Chacabuco	Buenos Aires	-34,642247	-60,852295		CH	
7	Villegas	Buenos Aires	-35,056980	-63,006592		GV	
8	Rafaela	Santa Fé	-31,269161	-61,484985	B	RF	
9	CABA	Buenos Aires	-34,590259	-58,457565		Q	
10	Rojas	Buenos Aires	-34,198173	-60,731049			RJ
11	Pergamino	Buenos Aires	-33,897777	-60,571060			PR
12	Oliveros	Santa Fé	-32,578063	-60,853958			OL
13	Pincen	Córdoba	-34,834096	-63,923950			W, WN
14	Areco	Buenos Aires	-34,265161	-59,449768	S		
15	Parana	Entre Ríos	-31.866785	-60.483346	PR		

*letters are reference for each collecting event
