# Supplementary material for: Characterized non-transient microbiota from stinkbug (Nezara viridula) midgut deactivates soybean chemical defenses
Source: PLoS One. 2018 Jul 12;13(7):e0200161. doi: 10.1371/journal.pone.0200161 (PMC6042706; doi:10.1371/journal.pone.0200161)
Supplement: S2 Table — (PDF) [file pone.0200161.s002.pdf]

S2 Table. Bacterial communities in the midgut of *Nezara viridula* associated to the insects hosts.

Nº[*1]		Insect host	Date	Ref[*2]	n[*3]	NTM[*4]	TM[*5]	NON-TRANSIENT MICROBIOTA													TRANSIENT MICROBIOTA				
								***Yokenella* sp**			***Enterococcus* sp**		***Cedecea* sp**		***Pantoea* sp**			Caeca symbiont		***Bacillus* sp**	***Micrococcus* sp**	***Streptomyces* sp**	***Staphylococcus***	**Others[*8]**
								ITS lengths (bp)[*6] // CFU/mg gut[*7]																
								626	710	780	502	602	659	815	651	667	858	748	756	ND	ND	ND	ND	ND
1	Secondary host	*Morus nigra*	30/09/12	H	8	4	0	6e5			2e6													
2	Secondary host	*Passiflora* sp	30/09/14	A	4	3	0	1e6																
3	Secondary host	*Triticum aestivum*	13/10/14	B	10	0	3													<100	<100			<100
4	Secondary host	*Brassica napus*	25/10/12	S	4	1	2				5e5										<10			
5	Secondary host	*Acacia megaloxylon*	18/01/12	J	8	0	2													<100	<100	<10	<100	
6	Secondary host	*Arcticum lapa*	24/01/12	I	8	0	3													<100	<100	<10	<100	<10
7	Secondary host	*Caria Illinoinensis*	12/03/13	C	2	0	0																	
8	Secondary host	*Zea mays*	24/02/14	PR	8	0	4																	
9		***Glycine max.***	06/02/13	T	4	0	0																	<10
10			19/03/12	M	4	3	2	2e5			8e6									<100	<10			
11			24/03/12	O	4	2	1	8e6												<100				
12			07/04/14	MJ	8	4	0						2e4											
13			11/04/14	CH	8	0	0																	
14			18/04/14	GV	5	0	0																	
15			24/04/12	P	6	2	0	3e6							1e4									
16			29/04/14	RF	10	0	0																	
17			13/06/12	Q	4	0	1													<100	<10			
18		***Eucalyptus* sp (Diapause)**	25/05/14	RJ	10	4	0	3e6																
19			24/06/13	PL	4	0	0																	
20			24/06/13	U	4	3	0	2e6																
21			05/07/14	PG	10	0	0																	
22			17/07/12	R	8	2	0	3e4																
23			19/07/14	OL	10	2	0								4e5									
24			04/08/13	W	6	2	0	3e4			3e5													
25			21/08/14	WN	6	2	0				1e2													
26			03/09/14	PA	10	3	0	3e4																

*1. Collecting event order by day and month in the year cycle of *Nezara viridula*. *2. Reference of collecting event used also to identify isolated strains. *3. Number of stinkbug adults analyzed. *4. NTM: number of stinkbug that presented Non-Transient microbiota infected midguts. *5. TM: number of stinkbugs that presented transient microbiota infected midguts. *6. Bacterial 16S-23S rRNA Intergenic Spacer (ITS) lengths identified in ARISA profiles. *7. Colony Forming Units per mg stinkbug midgut obtained on Triptein Soy Agar plates. *8. Other Transient microbiota (TM) species that were not thoroughly identified.
