# Supplementary material for: Characterized non-transient microbiota from stinkbug (Nezara viridula) midgut deactivates soybean chemical defenses
Source: PLoS One. 2018 Jul 12;13(7):e0200161. doi: 10.1371/journal.pone.0200161 (PMC6042706; doi:10.1371/journal.pone.0200161)
Supplement: S3 Table — (PDF) [file pone.0200161.s003.pdf]

S3 Table. Bacteria isolated in this work and those used to build phylogenetic trees of Yokenella.

Label*1	Specie*2	Culture collection ID*3	MALDI TOF score	Type strain	Gene Bank ID*4	Reference	Stinkbug host
**NvH01**	*Yokenella* sp	BNM 0559	2,04	No	KJ397957	This work	*Morus nigra*
**NvP02**	*Yokenella* sp	BNM 0559	1,73	No	KR537289	This work	*Glycine max.*
**NvO01**	*Yokenella* sp	BNM 0557	2,07	No	KJ397958	This work	*Glycine max.*
**NvW01**	*Yokenella* sp	BNM 0549	1,96	No	KR537290	This work	*Glycine max.*
**NvU01**	*Yokenella* sp	BNM 0558	1,94	No	KJ397960	This work	*Eucalyptus* trees (Diapause)
**NvU02**	*Yokenella* sp	BNM 0553	1,85	No	KJ397959	This work	*Eucalyptus* trees (Diapause)
**NvR01**	*Yokenella* sp	BNM 0552	1.96	No	KR537288	This work	*Eucalyptus* trees (Diapause)
**ATCC 49455**	Y. *resgensburgei*	ATCC 49455	-	Yes	JN175339	ATCC	-
**M-T-MRS 67**	Y. *resgensburgei*	M-T-MRS 67	-	No	JQ795833		-
**Hirose 2006**	K. *pneumoniae*	-	-	No	AY830394	Hirose et al, 2006	Lab reared
**Hirose 2006**	K. *pneumoniae*	-	-	No	AY830395	Hirose et al, 2006	Lab reared
**ATCC 13182**	*K. oxytoca*	ATCC 13182	-	Yes	AF129440	ATCC	-
**ATCC BAA-2403**	*K. michiganensis*	ATCCBAA-2403	-	Yes	JQ070300	ATCC	-
**ATCC 13885**	*K pneumoniae*	ATCC 13885	-	Yes	AF130981	ATCC	-

*1: Label used in the phylogenetic tree. *2: Specie denomination identified through 16S rRNA sequenciation (1450pb). *3: Culture collection denomination. *4. Accession number of sequences deposited in Genebank.
