# Supplementary material for: Characterized non-transient microbiota from stinkbug (Nezara viridula) midgut deactivates soybean chemical defenses
Source: PLoS One. 2018 Jul 12;13(7):e0200161. doi: 10.1371/journal.pone.0200161 (PMC6042706; doi:10.1371/journal.pone.0200161)
Supplement: S4 Table — (PDF) [file pone.0200161.s004.pdf]

S4 Table. Bacteria isolated in this work and those used to build phylogenetic trees of *Enterococcus* sp.

Label*[1]	Specie*[2]	Culture collection ID*[3]	Type strain	Gene Bank ID*[4]	Reference	Stinkbug host
**NvM04**	*Enterococcus* sp.	BNM 0551	No	KR537287	This work	*Glycine max.*
**NvS01**	*Enterococcus* sp.	BNM 0550	No	KJ397965	This work	*Brassica napus*
**NvW02**	*Enterococcus* sp	BNM 0555	No	KJ397963	This work	*Glycine max*
**NvH02**	*Enterococcus* sp.	BNM 0556	No	KJ397964	This work	*Morus nigra*
**Clone 1-1**	*Enterococcus* sp.	-	No	AY830397	Hirose et al (2006)	Lab reared
**Clone 1-4**	*Enterococcus sp*	-	No	AY830399	Hirose et al (2006)	Lab reared
**JCM 5803**	*E. faecalis*	JCM 5803	Yes	AB012202	ATCC	-
**ATCC BAA-383**	*E. moriaviensis*	ATCC BAA-383	Yes	AF286831	ATCC	-

*1: Label used in the phylogenetic tree. *2: Specie denomination identified through 16S rRNA sequenciation (1450pb). *3: Culture collection denomination. *4. Accession number of sequences deposited in Genebank.
