# Supplementary material for: Characterized non-transient microbiota from stinkbug (Nezara viridula) midgut deactivates soybean chemical defenses
Source: PLoS One. 2018 Jul 12;13(7):e0200161. doi: 10.1371/journal.pone.0200161 (PMC6042706; doi:10.1371/journal.pone.0200161)
Supplement: S1 Fig — Nezara viridula adults were handpicked from secondary hosts (light grey spots), Soybean (dark grey spots) or from under de bark of Eucalyptus trees (black spots). (PDF) [file pone.0200161.s005.pdf]

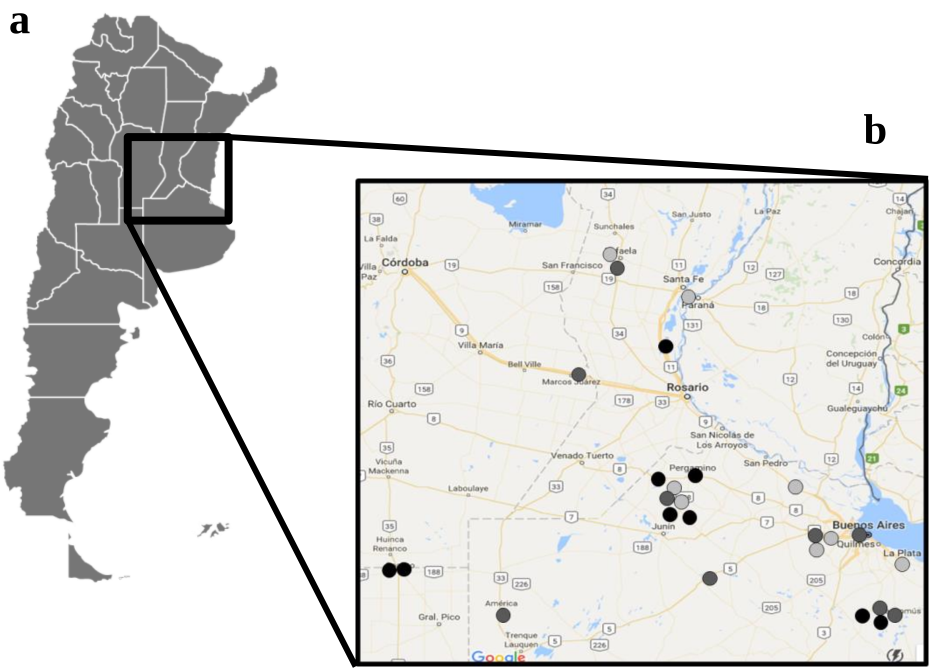

S1 Figure. **Map of Argentina (a) and a zoom of Central east Argentina (b) were 26 collecting events were performed during 2012-2014**. *Nezara viridula* adults were handpicked from secondary hosts (light grey spots), Soybean (dark grey spots) or from under de bark of Eucalyptus trees (black spots).
