# Supplementary material for: Characterized non-transient microbiota from stinkbug (Nezara viridula) midgut deactivates soybean chemical defenses
Source: PLoS One. 2018 Jul 12;13(7):e0200161. doi: 10.1371/journal.pone.0200161 (PMC6042706; doi:10.1371/journal.pone.0200161)
Supplement: S2 Fig — (a) Cysteine protease activity of N. viridula V1-V4 midgut ventricles. Statistical differences are denoted by different letters. (b) Distribution of ARISA detected bacteria among N. viridula V1-V4 midgut ventricles. Bacterial ITS fragments appear as blue peaks and LIZ 1200 weight standard fragments appear as yellow peaks. On a black square are 748 y 756bp cloacae symbiont ITS fragments. Numbers are reference for weight standard. (PDF) [file pone.0200161.s006.pdf]

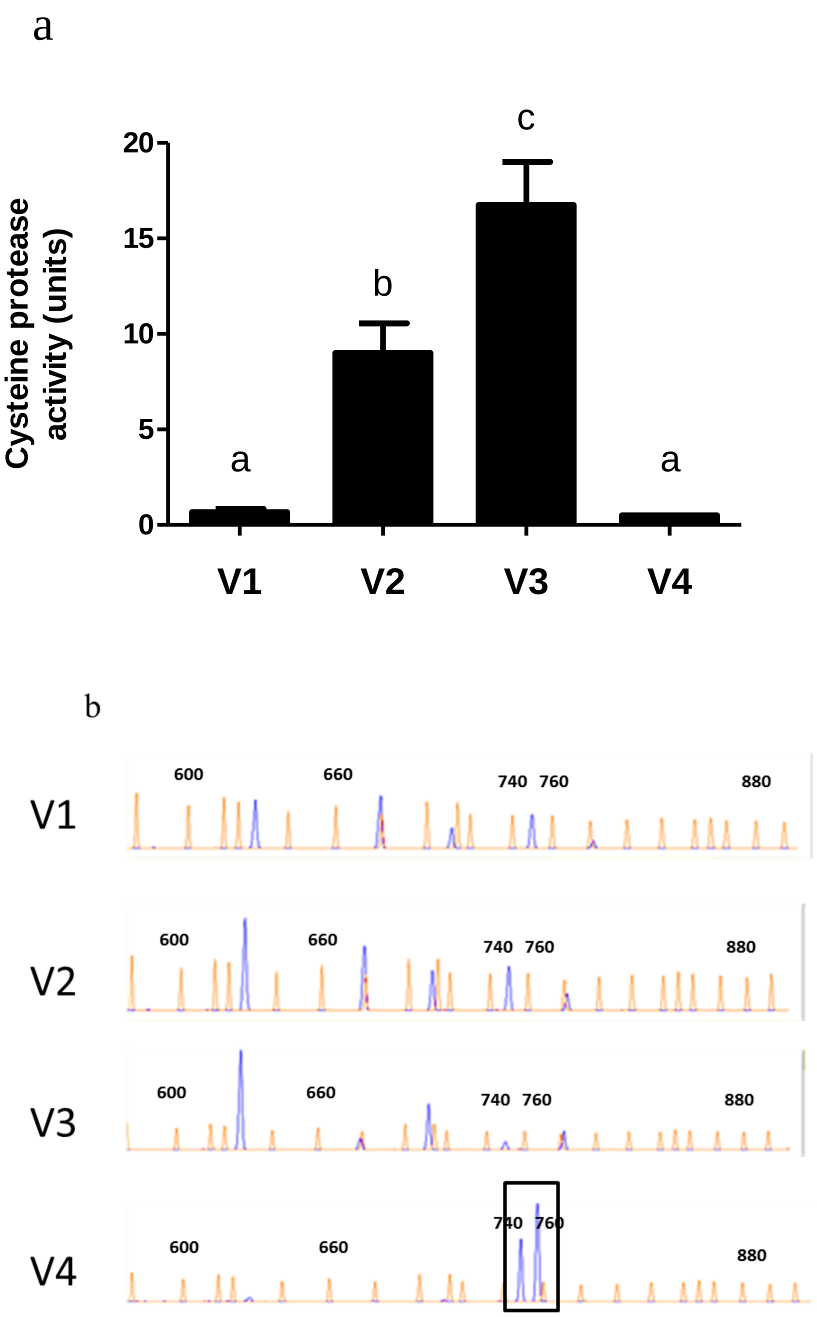

S2 Figure. (a) Cysteine protease activity of *N. viridula* V1-V4 midgut ventricles. Statistical differences are denoted by different letters. (b) Distribution of ARISA detected bacteria among *N. viridula* V1-V4 midgut ventricles. Bacterial ITS fragments appear as blue peaks and LIZ 1200 weight standard fragments appear as yellow peaks. On a black square are 748 y 756bp cloacae symbiont ITS fragments. Numbers are reference for weight standard.
